# Chromosome-level genome assembly assisting for dissecting mechanism of anthocyanin regulation in kiwifruit (*Actinidia arguta*)

**DOI:** 10.1186/s43897-024-00139-7

**Published:** 2025-04-01

**Authors:** Yukuo Li, Zhe Song, Xu Zhan, Xiaohan Li, Lingshuai Ye, Miaomiao Lin, Ran Wang, Hailei Huang, Jian Guo, Leiming Sun, Hong Gu, Jinyong Chen, Jinbao Fang, Xiujuan Qi

**Affiliations:** 1 National Key Laboratory for Germplasm Innovation & Utilization of Horticultural Crops, Zhengzhou Fruit Research Institute, Chinese Academy of Agricultural Sciences, Zhengzhou, 450009 China; 2 Zhongyuan Research Center, Chinese Academy of Agricultural Sciences, Xinxiang, 453500 China; 3 School of Agricultural Sciences, Zhengzhou University, Zhengzhou, 450001 China; 4 College of Horticulture and Plant Protection, Henan University of Science and Technology, Luoyang, 471000 China; 5 Shiyan Economic Crops Research Institute, Shiyan, 442099 China; 6 College of Horticulture Science and Engineering, Shandong Agricultural University, Tai’an, 271018 China

**Keywords:** Genome assembly, Comparative genome, Gene expression, Anthocyanin biosynthesis, *Actinidia arguta*

## Abstract

*Actinidia arguta* is a newly emerged, commercially cultivated *Actinidia* species. *A. arguta* has a beautiful appearance and is rich in anthocyanin, and is thus highly welcomed by consumers. However, the mechanism of anthocyanin regulation in *A. arguta* remains unclear. In this study, we assembled the nearly complete genome of the first red *A. arguta* cultivar, ‘Tianyuanhong’, with an N50 of 21 Mb. Comparative genome analysis revealed a role of the expansion/contraction of gene families in the species-specific trait formation of *A. arguta*. Through verification of transient overexpression and stable transformation, RNA-seq analysis revealed a key bHLH transcription factor, AaBEE1, which negatively regulates anthocyanin biosynthesis. DAP-seq analysis combined with Y1H, EMSA, Chip-qPCR and LUC suggested that AaBEE1 binds to the G-box of the *AaLDOX* promoter and suppresses its expression. Overall, we assembled the genome of *A. arguta* and clarified its AaBEE1-AaLDOX module-mediated molecular mechanism of anthocyanin regulation.

**Supplementary Information:**

The online version contains supplementary material available at 10.1186/s43897-024-00139-7.

## Core

We assembled a high-quality chromosome-level genome of red *A. arguta* ‘Tianyuanhong’, and analyzed comparative genome and transcriptome as well as various molecular biology investigation to reveal a candidate TF AaBEE1, that negatively regulates anthocyanin biosynthesis by directly targeting the *AaLDOX* promoter, in which a 29-bp indel variation associated with fruit color was identified, and an indel marker was developed for color breeding.

## Gene & Accession Numbers

All sequence data and genome files generated for this study were deposited in NGDC (https://ngdc.cncb.ac.cn/gsa/) under Bio Project ID PRJCA033191.

## Introduction

Kiwifruit is an important fruit vine that originated in China and belongs to the genus *Actinidia*, which comprises 54 species and 21 varieties (Huang 2013; Fang and Zhong 2019). Kiwifruit color is mainly green, yellow or red, and commercially cultivated *A. chinensis var. chinensis* or *A. chinensis var. deliciosa* varieties are mainly green, yellow or radiant red. Recently, red *A. arguta* has become popular on the market owing to its attractive appearance, moderate size, edible skin, and readiness for consumption immediately after harvest; in particular, its rich anthocyanins cause both its skin and flesh to be red, unlike the traditional radiant red *A. chinensis* (also called red-core kiwifruit), which causes red *A. arguta* to be loved by consumers. However, the red coloration of currently bred *A. arguta* cultivars is generally unstable after cultivation. While identifying key variations and genes underlying the molecular mechanism of anthocyanin regulaion is the molecular basis for breeding red fruit, this mechanism in *A. arguta* remains unclear.

Genetic variations associated with red color have been shown to be involved in anthocyanin regulation in other fruit trees. Example variations include multiple repeats of the 23-bp R motif of the MdMY10 promoter in red-fleshed apple (Espley et al. 2009), the *Tcs1* retrotransposon of the *CsRuby1* promoter in red-fleshed orange (Butelli et al. 2012; Huang et al. 2018), and *CACAT-like* transposon of the *FaMYB10* promoter in cultivated strawberry (Castillejo et al. 2020), which play regulatory roles in the anthocyanin pathway by affecting the expression of relevant genes. In kiwifruit, AcMYB10, AcMYB75, AcMYB110, AcMYBF110 and AcMYB123 regulate anthocyanin biosynthesis by activating the expression of anthocyanin biosynthesis genes, including *AcDFR*, *AcF3H*, *AcLDOX* and *AcF3GT1* (Liu et al. 2017; Li et al. 2017; Wang et al. 2019; Yu et al. 2019). Wang et al. reported that *A. chinensis* AcMYB10 and AcMYB110 might have originated from the same ancestor and subfunctionalized during the evolutionary process, which caused the specific expression of *AcMYB10* and *AcMYB110* in red heart flesh and whole red flesh, respectively (Wang et al. 2022b). AaMYB110 also participated in the regulation of the anthocyanin pathway in *A. arguta* (Peng et al. 2019). In a previous study, we reported that R2R3-MYB AaMYBC1 regulates anthocyanin biosynthesis by interacting with AabHLH42 (Li et al. 2020). AaMYBC1 was subsequently confirmed to dynamically regulate anthocyanin and proanthocyanidin together with AaWRKY44, thus maintaining the balance of flavonoid levels (Peng et al. 2020), which provides useful information for elucidating the mechanism of color formation in kiwifruit. These TFs have been identified as functional regulators involved in anthocyanin biosynthesis but are rarely used for marker development due to a lack of key color-related variation.

A high-quality chromosome-scale genome is the cornerstone for gene mining and variation identification. Since the release of the first draft genome of *A. chinensis* ‘Hongyang’ (Huang et al. 2013), there more than 10 versions of the kiwifruit genome have been published, including *A. chinensis*, *A. eriantha*, *A. latifolia*, *A. zhejiangensis*, *A. hemsleyana*, *A. polygama*, and *A. rufa* (Akagi., 2023; Han et al. 2023; Liao et al. 2023; Pilkington et al. 2018; Tang et al. 2019; Wu et al. 2019; Wang et al. 2023b; Wang et al. 2024; Xia et al. 2023; Yu et al. 2023; Yue et al. 2022, 2023), which provide an important basis for functional genomics studies. Recently, a pangenome study of seven *A. chinensis* cultivars revealed that indel variation in the *BCM* promoter plays a crucial role in green fruit color formation (Wang et al. 2024). In *A. arguta*, a 135 K SNP genotyping array provides possible applications for genetic mapping and QTL analysis (Wang et al. 2023a). Furthermore, the *A. arguta* green female cultivar ‘LC2’ and male cultivar ‘M1’ were assembled to explore their evolutionary history and sex divergence (Zhang et al. 2024; Lu et al. 2024). However, the lack of a red female *A. arguta* chromosome-level genome limits the ability to mine anthocyanin-related genes.

In this study, we assembled the first chromosome-level reference genome for *A. arguta* with all red flesh, on the basis of which we performed comparative genome and RNA-seq analyses and identified AaBEE1 as a negative regulator of anthocyanin biosynthesis. Further DAP-seq combined with various molecular biological methods revealed that the AaBEE1-*AaLDOX* module mediates the anthocyanin regulatory mechanism. Notably, a 29-bp indel variation in the *AaLDOX* promoter was found to be highly linked with the color phenotype and was used as an indel marker for color breeding.

## Results

### Genome assembly of *A. arguta* ‘Tianyuanhong’ and comparative genome analysis

**Fig. 1:**
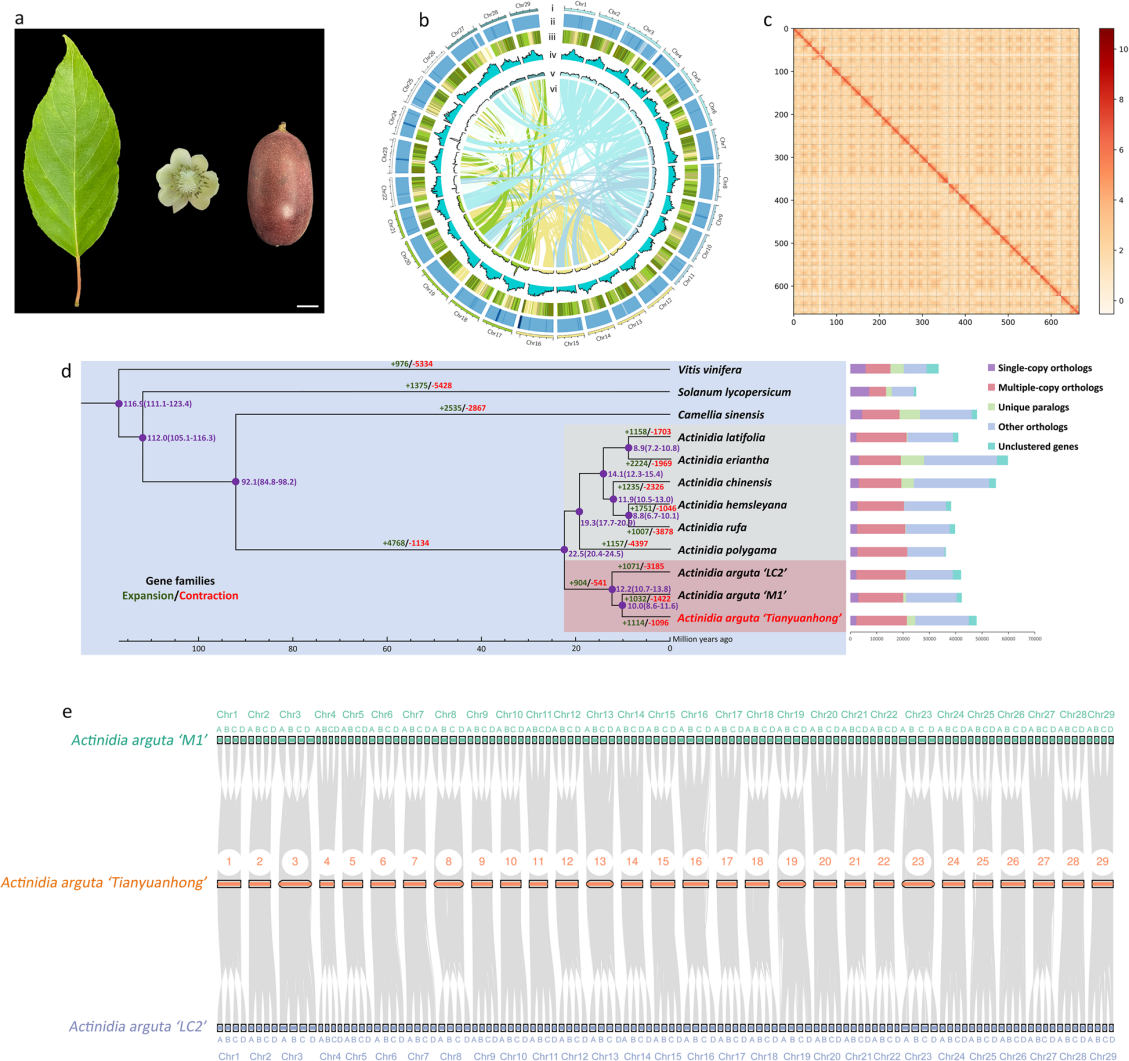
Genome assembly and comparative genome analysis. **a** Phenotype of *A. arguta* leaf, flower and fruit. Scale bar: 1 cm. **b** Distribution of *A. arguta* genomic features. Tracks i-vi represent the 29 chromosomes, GC content, gene density, distribution of repetitive sequences, distribution of DNA transposons, and collinearity of different chromosomes, respectively. **c** Hi-C interaction heatmap for the *A. arguta* genome. **d** Phylogenetic tree, species divergence, gene family analyses, and gene distribution. **e** Genome synteny of the ‘Tianyuanhong’ genome with *A. arguta* ‘M1’ and *A. arguta* ‘LC2’

The red *A. arguta* cultivar ‘Tianyuanhong’ was selected from Funiu Mountain and bred. We sequenced the genome of ‘Tianyuanhong’ via the PacBio Revio sequencing platform. A total of 45.05 G PacBio HiFi long reads were generated, and de novo assembly of the HiFi reads yielded 815.19 Mb consensus contigs with an N50 value of 20.14 Mb. After combination with 60 G Hi-C data, a total of 693.43 Mb contigs (95.93% of the assembly) with an N50 of 21.0 Mb were successfully clustered into 29 chromosomes. A Hi-C interaction heatmap revealed 29 superscaffolds in the *A. arguta* genome could be distinguished and perfectly represented by 29 chromosomes (Fig. 1a-c), among which 20 had no gaps (Fig. S1). By integrating homology-based prediction, full-length transcriptome data from RNA-seq/Iso-seq and de novo prediction, a total of 47,899 protein-coding genes were predicted. Compared with published genomes, such as the 616.2 Mb genome of *A. chinensis* with 45,809 protein-coding genes, the 640.56 Mb genome of *A. latifolia* with 41,317 protein-coding genes, and the 615.95 Mb genome of *A. eriantha* with 35,530 protein-coding genes, the *A. arguta* genome is larger and has more genes, possibly resulting from gene expansion during the homologous tetraploidization process (Table S1). Among the 47,899 genes, 45,169 were successfully annotated in gene functional databases, accounting for 94.3% of the genome (Table S2). Repeat sequences are important components of the genome. A total of 367.84 Mb of repeat sequences, accounting for 53.05% of the genome, were detected (Table S3), in which transposons predominated and comprised 48.63% of the genome. Long terminal repeat (LTR) transposons were the main type of transposons and accounted for 25.54% of the genome (Table S4). In addition, non-coding RNAs, including miRNAs, tRNAs, rRNAs and snRNAs, comprised 2.89% of the genome (Table S5). The genome size of *A. arguta* was estimated to be approximately 2650.01 Mb based on k-mer analysis, significantly exceeding that reported for the diploids *A. chinensis* (Han et al. 2023) and *A. eriantha* (Liao et al. 2023). In addition, the k-mer curve of *A. arguta* was autotetraploid (Fig. S2), similar to that of other reported autotetraploid species, such as *R. officiale* (Zhang et al. 2023), *S. tuberosum* (Sun et al. 2022; Zhang et al. 2022a) and *M. sativa* (Chen et al. 2020). Five approaches were used to assess the quality of the *A. arguta* genome assembly. First, the resequenced short reads were mapped to the assemblies, reaching a 99.94% coverage rate. Second, the HiFi reads were also mapped to the assemblies, reaching a 99.97% coverage rate. Third, Benchmarking Universal Single-Copy Orthologs (BUSCO) evaluation was performed, resulting in a score of 98.9%. Fourth, assessment by Merqury showed a consensus quality (QV) value of 50.49. Fifth, the LTR Assembly Index (LAI) value was found to be over 16 (Table S1), indicating that the quality of the ‘Tianyuanhong’ assembly reached the reference level. Among 29 chromosomes, 26 had telomeric sequences (‘CCCTAAA’) at both ends, and the remaining 3 had telomeric sequences (‘CCCTAAA’) at only the forward end (Table S6). In addition, all 29 chromosomes had monomers (Table S6). All these parameters indicated that the genome assemble was nearly complete at the chromosome level.

The phylogenetic tree was constructed from 486 single-copy orthologs of 12 species, including *Vitis vinifera*, *Solanum lycopersicum*, *Camellia sinensis*, and 9 other *Actinidia* species. The *Actinidia* species presented a relatively close genetic relationship with *Camellia sinensis* species, and the diploid and tetraploid species diverged ~ 22.5 mya (Fig. 1d). The divergence time of tetraploid *A. arguta* was later than that of diploid *Actinidia* species, implying that tetraploids result from the doubling of diploid. Tetraploid *A. arguta* clustered into the same branch and presented high levels of genomic synteny with each other (Fig. 1e). *A. arguta* ‘Tianyuanhong’ diverged late, ~ 10.0 mya, which might be due to more gene family expansions and fewer gene family contractions than in *A. arguta* ‘M1’ and *A. arguta* ‘LC2’ (Fig. 1d). Gene Ontology (GO) enrichment results revealed that the expanded and contracted genes were enriched in both metabolic processes and cellular processes (Fig. S3a-b). Kyoto Encyclopedia of Genes and Genomes (KEGG) pathway enrichment results revealed that the expanded genes were associated with translation, environmental adaptation, metabolism of terpenoids and polyketides, biosynthesis of other secondary metabolites and transport and catabolism (Fig. S3c), whereas the contracted genes were associated with signal transduction (Fig. S3d). The expansion/contraction of gene families explain species-specific traits in *A. arguta*, including edible fruit skin without lignification, red fruit color when ripening, and stronger adaptations, such as cold tolerance and *Pseudomonas syringaepv.* actinidiae (PSA) resistance.

### Screening of the color-related transcription factor AaBEE1 via RNA-seq analysis

**Fig. 2:**
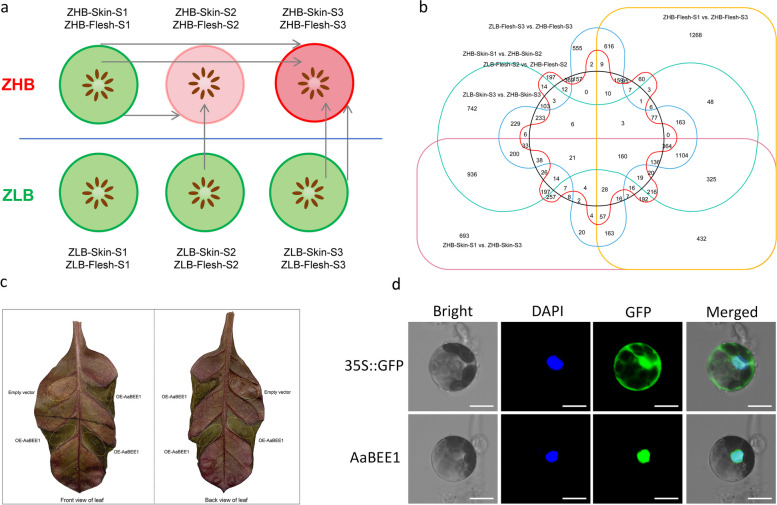
Comparative transcriptome analysis mining the candidate gene *AaBEE1*. **a** Two different color-type *A. arguta* cultivars, ‘ZHB’ and ‘ZLB’, at three development stages were used for transcriptome analysis. The solid arrows indicate comparisons between two samples. **b** Venn diagram of the six different comparisons used for gene screening. **c** Transient overexpression of the candidate gene *AaBEE1* in the red-background tobacco. **d** Subcellular localization of AaBEE1 in *Arabidopsis thaliana* protoplasts. Scale bar: 10 μm

Two tetraploid *A. arguta* cultivars, ‘ZHB’ with a red color in both the skin and flesh when the fruit ripens and ‘ZLB’ with a green color in both the skin and flesh when the fruit ripens, at three different stages were subjected to RNA-seq based on the ‘Tianyuanhong’ genome (Fig. 2a). The differentially expressed genes (DEGs) in the six comparisons indicated by solid arrows in Fig. 2a were analyzed. Given that ‘ZHB’ had the greatest difference at S1 and S3 both in skin and flesh during fruit development, we compared the fruit skin and flesh of ZHB-S1 to those of ZHB-S3. Considering that S3 is the stage when ‘ZHB’ and ‘ZLB’ had the greatest color differences in both skin and flesh, we compared the fruit skin and flesh of ZLB-S3 to those of ZHB-S3. ZHB-S2 is the color transition stage in the middle of the fruit coloring process, so we compared the skin of ZHB-S1 to that of ZHB-S2 and the flesh of ZLB-S2 to that of ZHB-S2, which resulted in a total of six comparisons to cover as much of the color phenotype process as possible. A total of 1521 DEGs were obtained (Fig. S4a), among which 299 were upregulated (Fig. S4b) and 160 were downregulated (Fig. 2b) by twofold (Table S8). Previous studies have focused on the positive regulators of kiwifruit color. To broaden our understanding anthocyanin regulation, we aimed to identify negative regulators. We identified candidate genes by comparing the above 160 downregulated DEGs with other downregulated DEGs whose expression changed eightfold. A total of 15 common downregulated DEGs were mined as candidate genes (Fig. S4c, Table S9). According to the functional annotation, we focused on Aar28009 annotated as transcript factor BEE, that has been shown to be a negative anthocyanin regulator in *Arabidopsis thaliana* (Petridis et al. 2016). We cloned the full-length CDS of Aar28009, which is 831 bp encoding 276 amino acid, from *A. arguta* and conducted phylogenetic analysis between Aar28009 and other bHLH members of the IIIf subgroup, which are generally known as regulators involved in anthocyanin biosynthesis (Montefori et al., 2015). Aar28009 clustered into the same branch as AtBEE1 (Fig. S5), so we designated Aar28009 as AaBEE1. The expression of AaBEE1 was high in ‘ZLB’ but low in ‘ZHB’; in particular, a significant difference in *AaBEE1* expression occurred at S3 when ‘ZLB’ and ‘ZHB’ presented significant color differences (Fig. S6), indicating that the expression of *AaBEE1* was negatively correlated with fruit coloration. To preliminarily assess the function of AaBEE1, we carried out transient overexpression in red-background tobacco, which showed significant suppression after over-expressing AaBEE1 (Fig. 2c), revealing that AaBEE1 is a negative regulator involved in the anthocyanin pathway. The subcellular localization of AaBEE1 in *A. thaliana* protoplasts revealed that the green fluorescence of AaBEE1-GFP (Green fluorescent protein)-overexpressing plants was only in the nucleus (Fig. 2d), further indicating that AaBEE1 is a TF.

### Functional validation of AaBEE1

**Fig. 3:**
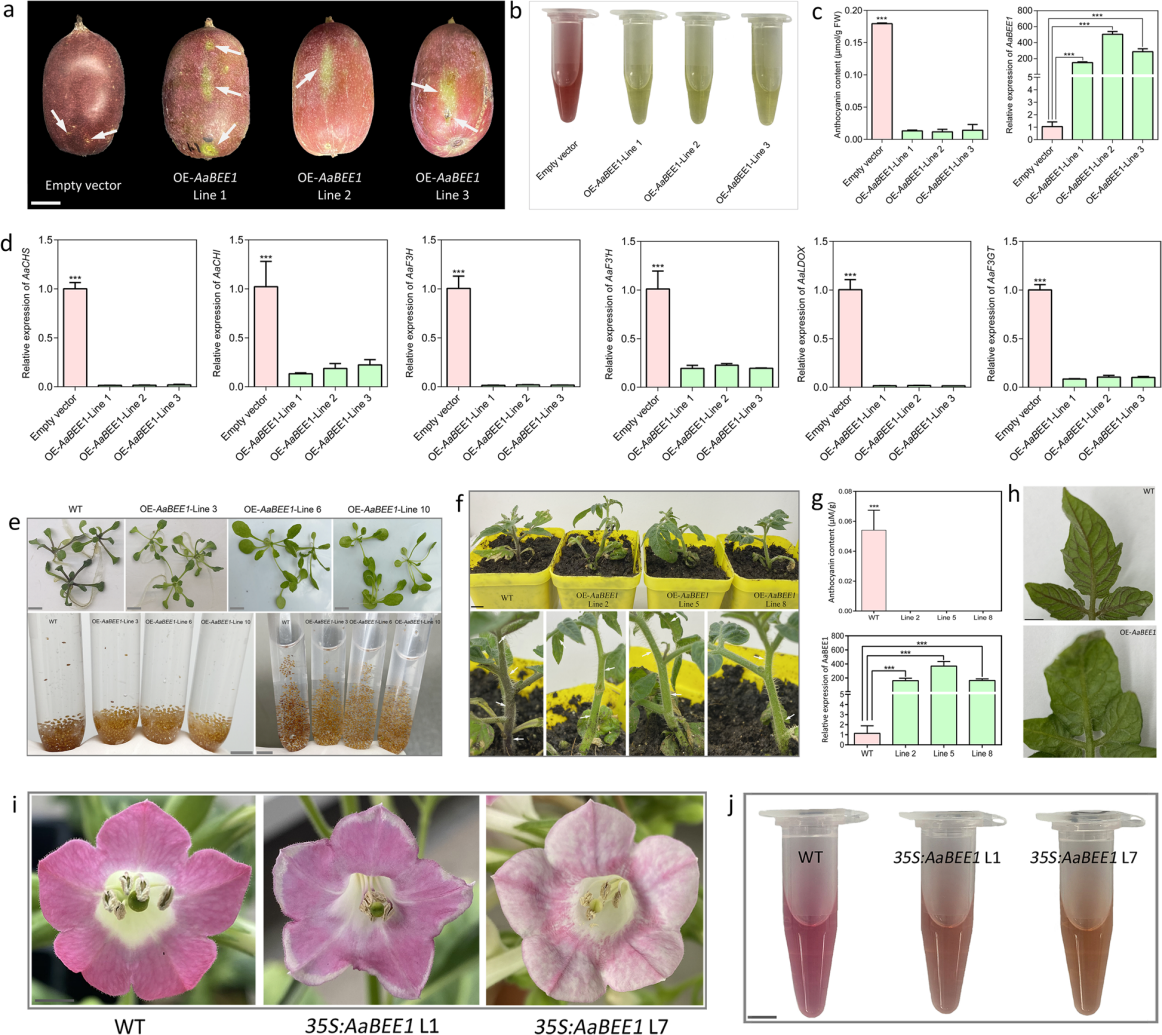
Functional validation of *AaBEE1*. **a** Transient overexpression of AaBEE1 in red *A. arguta*. The white solid arrows point to the infiltration areas. **b**, **c** Anthocyanin extracts, anthocyanin content and *AaBEE1* expression in samples infiltrated with the empty vector, OE-AaBEE1-Line 1, OE-AaBEE1-Line 2 or OE-AaBEE1-Line 3. The values are the means ± SDs for three replicates. Statistical significance: ***P < 0.001. **d** Relative expression of anthocyanin biosynthetic genes in the samples infiltrated with the empty vector, OE-AaBEE1-Line 1, OE-AaBEE1-Line 2 or OE-AaBEE1-Line 3. The values are the means ± SDs of three replicates. Statistical significance: ***P < 0.001. **e** T2 transgenic seeds were obtained via stable genetic transformation of *AaBEE1* in WT *A. thaliana* ‘Col-0’. The seedlings were observed one week after sowing on MS media. Seed coat color was also observed in the centrifuge tube. **f** Stable genetic transformation of *AaBEE1* in *S. lycopersicum* ‘Micro-Tom’. Overall growth state of the WT and three independent transgenic lines, namely, lines 2, 5 and 8, is shown above. The magnification is shown below. The white solid arrows point to positions with color differences. Scale bar: 1 cm. **g** Anthocyanin content and expression level of *AaBEE1* in the WT and three transgenic lines. The values are the means ± SDs of three replicates. Statistical significance: ***P < 0.001.** h** Leaf vein phenotypes of the WT and transgenic lines. Scale bar: 1 cm. **i** Flower color of WT tobacco and two independent transgenic tobacco lines, namely, line 1 and line 7. WT represents the wild type. L1 and L7 represent Line 1 and Line 7, respectively. Scale bar: 1 cm. **j** Anthocyanin extracts from WT and transgenic lines. Scale bar: 0.5 cm

To comprehensively confirm the function of AaBEE1 in anthocyanin biosynthesis, we first carried out transient overexpression in red *A. arguta* fruits. Compared with those infiltrated with the empty vector (EV), the specific fruit areas enriched with OE-AaBEE1 were not able to accumulate red pigments (Fig. 3a), and the anthocyanin extracts and contents were consistent with the phenotype (Fig. 3b-c). The expression of *AaBEE1* in OE-AaBEE1 was greater than that in EV (Fig. 3c), and the expression of most anthocyanin biosynthetic genes, including *AaPAL*, *AaCHS*, *AaCHI*, *AaF3H*, *AaF3’H*, *AaLDOX* and *AaF3GT*, was suppressed after the overexpressing of *AaBEE1* (Fig. 3d, Fig. S7). We subsequently conducted stable genetic transformation in different species, including *A. thaliana*, *S. lycopersicum* and *N. tabacum*. In *A. thaliana*, the seed coat color of the T2 transgenic lines was lighter than that of the WT, and the stems of one-week-old seedlings did not appear red from the base to the cotyledons (Fig. 3e). The anthocyanin content was lower in the transgenic lines than in the WT (Fig. S8a), and the expression of *AaBEE1* was greater in the transgenic lines than in the WT (Fig. S8b). The expression of structural genes, particularly *AtLDOX,* was inhibited in transgenic *Arabidopsis* (Fig. S9a). In *S. lycopersicum*, stems and leaf veins were purple in the WT lines but green in the transgenic lines (Fig. 3f, h). Anthocyanins were detected in the WT but not in the transgenic lines (Fig. 3g), and the expression of *AaBEE1* in the transgenic lines was several hundred times greater than that in the WT (Fig. 3g). The expression of structural genes, particularly *SlLDOX,* was inhibited in the transgenic tomato plants (Fig. S9b). In *N. tabacum*, the red pigments of the flowers of the transgenic lines were significantly attenuated compared with those of the WT plants (Fig. 3i). The anthocyanin extracts presented a similar phenotype in which the red color was lighter in the transgenic lines than in the WT plants (Fig. 3j). The expression level of *AaBEE1* in the transgenic lines was several thousand times greater than that in the WT (Fig. S10a). The expression of anthocyanin biosynthetic genes, including *Nt4CL*, *NtCHS*, *NtF3H* and *NtLDOX*, was lower in the transgenic lines than in the WT (Fig. S10b-f). Taken together, these data indicate that high expression of *AaBEE1* suppresses anthocyanin biosynthesis and accumulation, mainly by affecting the expression of structural genes.

### Identification of potential target genes of AaBEE1

**Fig. 4:**
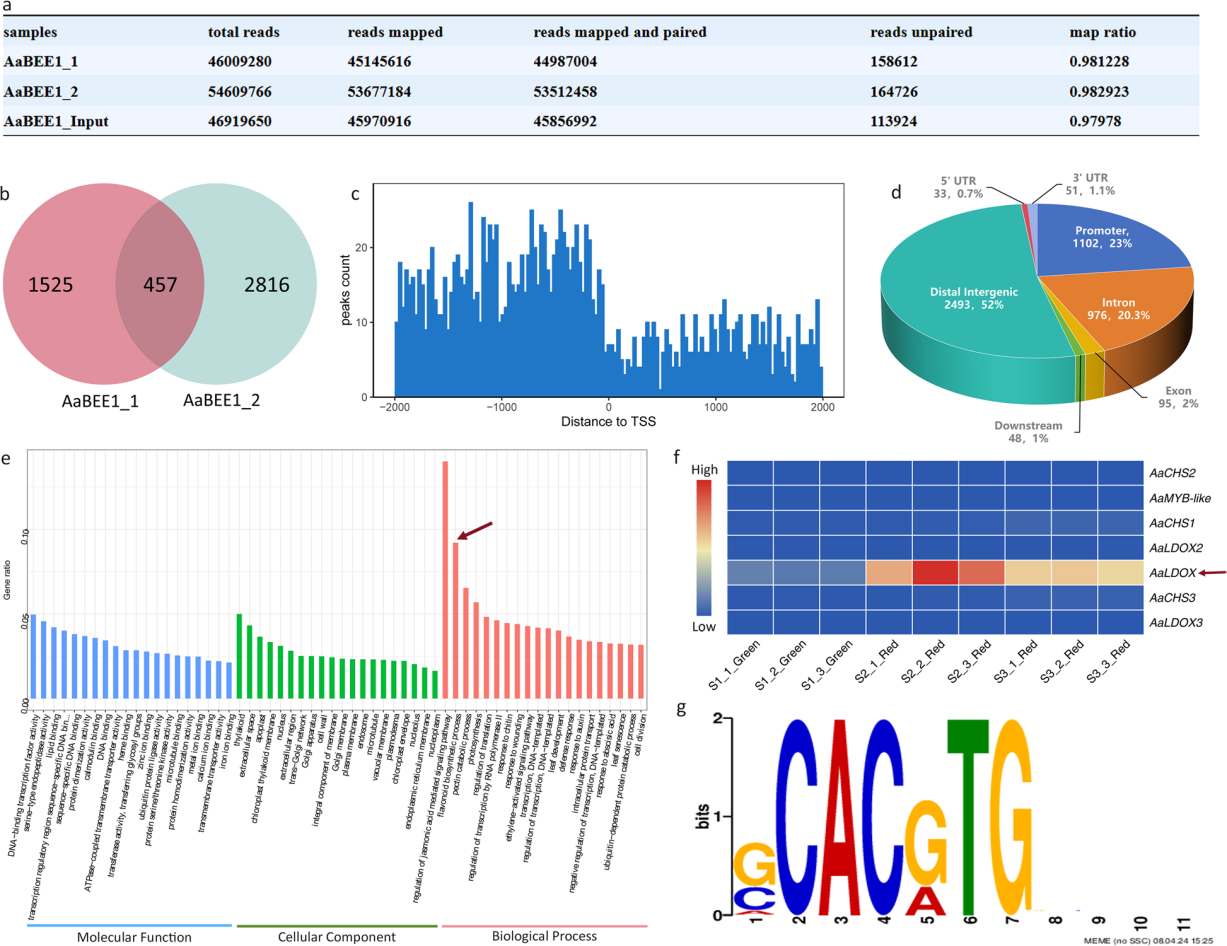
DAP-seq analysis identifying potential target genes of AaBEE1. **a** Statistics of sequence mapping. **b** Venn diagram of merged peaks. DAP-Seq experiments involving two biological replicates revealed 457 high-confidence AaBEE1 binding peaks.** c** Distribution of peak counts from binding sites to the TSS (transcription start site). **d** Statistics of binding sites within distal intergenic, intro, promoter, exon, downstream, 5’UTR (untranslated region) and 3’UTR regions. **e** GO enrichment of peak-associated genes. The abscissa represents three GO terms, namely, biological process, cellular component and molecular function. The ordinate represents the number of peak-associated genes. The solid red arrows point to the flavonoid biosynthetic pathway. **f** Expression profile of 7 genes involved in the flavonoid biosynthetic pathway. **g** Potential binding elements with the highest confidence level according to DAP-seq

To identify potential target genes of AaBEE1 in *A. arguta*, we performed DAP-seq of AaBEE1. Clean reads could be mapped to be reference genome at a 97% mapping ratio (Fig. 4a). A total of 457 AaBEE1-binding peaks across 29 chromosomes were obtained (Fig. 4b). The lengths of most binding peaks were distributed 2 kb upstream of the transcription initiation site (TSS) (Fig. 4c), which is usually the promoter region that accounts for 23% of the binding peaks (Fig. 4d). The binding peak-related target genes were subjected to GO enrichment analysis, in which the flavonoid biosynthetic process, which included 7 potential target genes, was enriched (Fig. 4e). *LDOX* (encoding leucoanthocyanidin dioxygenase) expression was highly correlated with fruit coloration (Fig. 4f). The motif G-box (CACGTG), identified as the highest scoring cis-acting element from DAP-seq, was predicted at the *AaLDOX* promoter (Fig. 4g), suggesting that *AaLDOX* might be the direct target of AaBEE1.

### *AaLDOX* is the direct target of AaBEE1

**Fig. 5:**
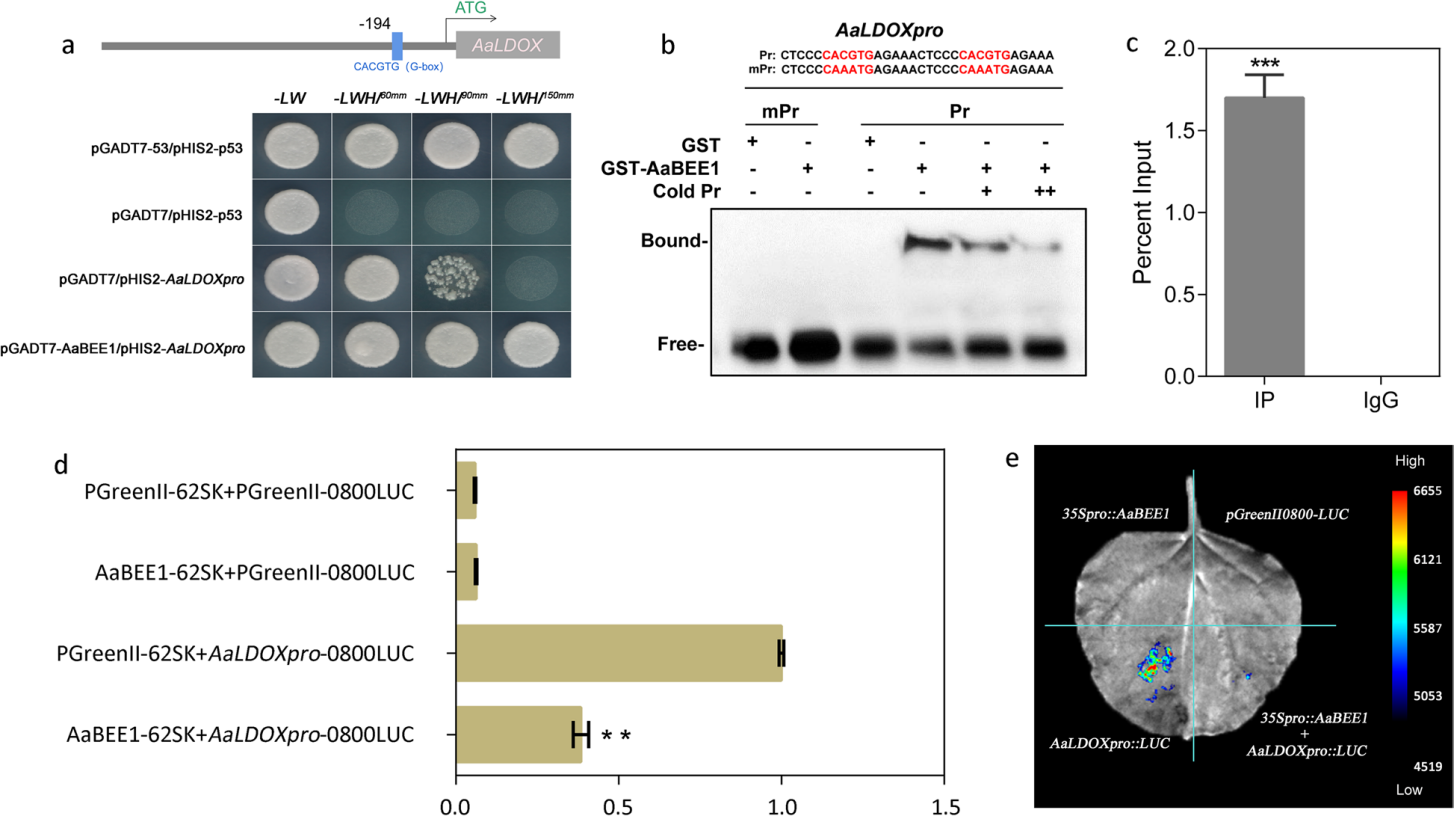
Binding of AaBEE1 to the *AaLDOX* promoter. **a** Yeast one-hybrid assay between AaBEE1 and the *AaLDOX* promoter. pGADT7-53/pHIS2-p53 and pGADT7/pHIS2-p53 were used as positive and negative controls, respectively. **b** Electrophoretic mobility shift assays revealed the direct binding of the GST-AaBEE1 recombinant protein to the G-box of the *AaLDOX* promoter. A mutated Probe (mPr), in which the G-box 5’-CACGTG-3’ was replaced with 5’-CAAATG-3’, was used as a control.** c** Chip-qPCR verification of AaBEE1 binding to the G-box of the *AaLDOX* promoter in *A. arguta* leaves expressing AaBEE1 with a GFP tag. The percent input method was selected for quantification via qPCR. IP represents the experimental group showing the specific enrichment. IgG represents the negative control. The values are the means ± SDs of three replicates. Statistical significance: ***P < 0.001. **d** Dual-luciferase assay of transient overexpression in *N. benthamiana* leaves coinjected with the AaBEE1 and *AaLDOX* promoters. The values are the means ± SDs of three replicates. Statistical significance: **P < 0.01. **e** Images of *N. benthamiana* leaves were captured 3 days after infiltration. Blue and red represent low and high luminescence intensities, respectively

To verify the binding of AaBEE1 to the *AaLDOX* promoter, we cloned promoter sequence of *AaLDOX* and identified the G-box element 194 bp upstream of the *AaLDOX* ATG (Fig. 5a). We carried out a yeast one-hybrid (Y1H) assay, which revealed that the Y187 yeast strain cotransformed with the pGADT7-*AaBEE1* and pHIS2-*AaLDOXpro* plasmids could grow on media supplemented with 150 mM 3-AT, whereas the yeast strain cotransformed with pGADT7 and pHIS2-*AaLDOXpro* could not, suggesting a possible interaction between AaBEE1 and the *AaLDOX* promoter (Fig. 5a). Electrophoretic mobility shift assays (EMSA) confirmed the direct binding of AaBEE1 to the G-box of the *AaLDOX* promoter by using a mutated probe, in which the G-box 5’-CACGTG-3’ was replaced with 5’-CAAATG-3’ as the control (Fig. 5b). To verify the interaction in vivo, a Chip-qPCR assay was carried out using cell extracts from *A. arguta* leaves expressing AaBEE1-GFP fusions, which revealed specific enrichment of AaBEE1 binding to the *AaLDOX* promoter (Fig. 5c). Next, we performed a dual-luciferase (LUC) assay in tobacco leaves. The Luc/Ren ratio in the plants cotransformed with AaBEE1-62SK and *AaLDOXpro-0800LUC* was lower than that in the control plants cotransformed with PGreenII-62SK and *AaLDOXpro-0800LUC* (Fig. 5d). Luciferase imaging revealed that tobacco leaves coinfiltrated with the AaBEE1 and *AaLDOX* promoters presented lower luminescence signals than did the control plants (Fig. 5e), indicating that AaBEE1 suppressed the activity of the *AaLDOX* promoter. Taken together, these findings indicate that AaBEE1 suppresses *AaLDOX* promoter activity by binding to the G-box, thus inhibiting *AaLDOX* expression.

### *AaLDOX* is the key structural gene involved in anthocyanin biosynthesis

**Fig. 6:**
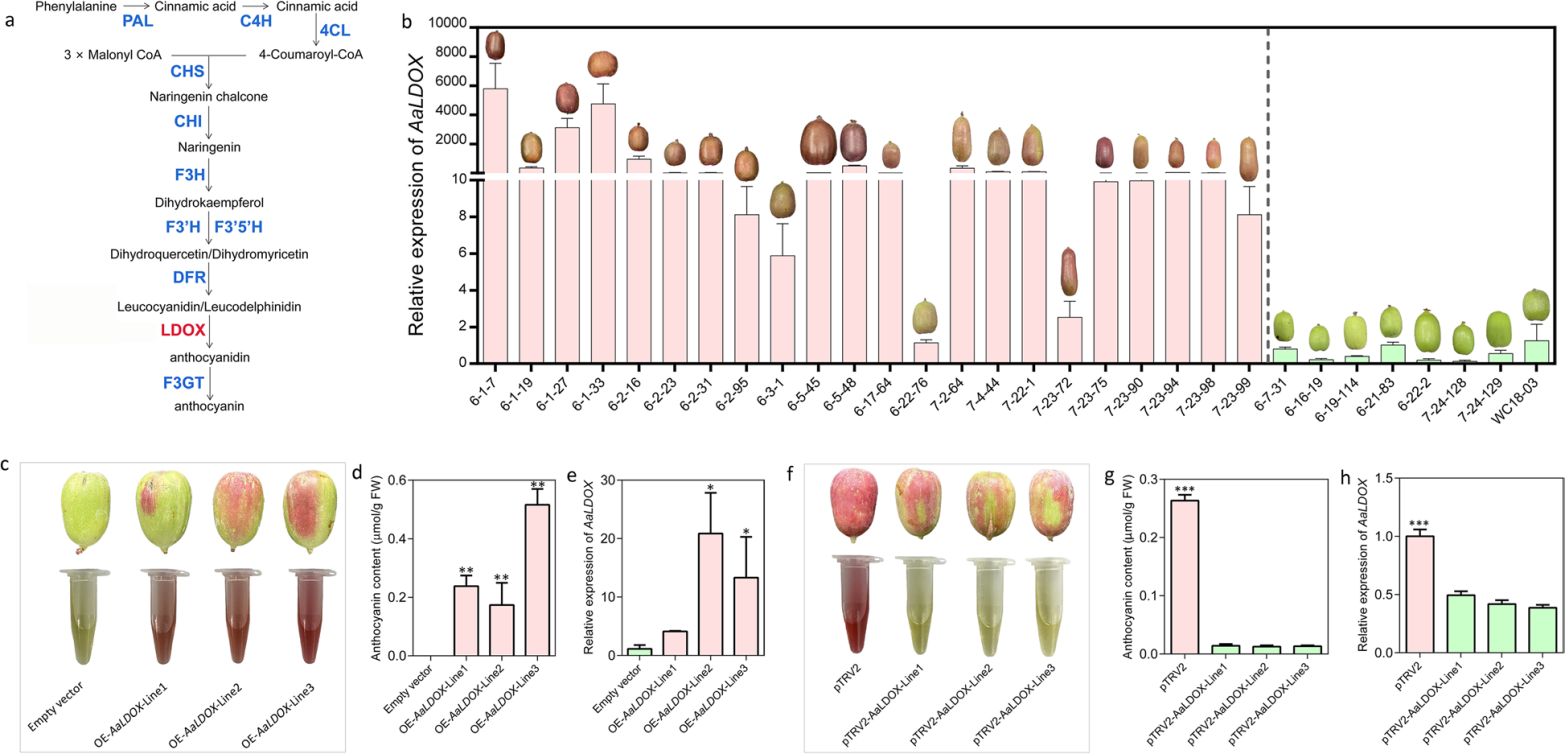
Functional validation of *AaLDOX*. **a** Anthocyanin biosynthetic pathway that requires the catalysis of a series of enzymes. PAL phenylalanine ammonia-lyase, C4H trans-cinnamate 4-hydroxylase, 4CL 4-coumarate:CoA ligase, CHS chalcone synthase, CHI chalcone isomerase, F3H flavanone 3-hydroxylase, F3’H flavonoid 3'-hydroxylase 2, F3′5’H flavonoid 3',5'-hydroxylase, DFR dihydroflavonol-4-reductase, LDOX leucoanthocyanidin dioxygenase, F3GT flavonoid 3-O-galactosyltransferase. **b** Relative expression level of *AaLDOX* in red and green *A. arguta* fruits. The values are the means ± SDs for three replicates. **c-e** Phenotype, anthocyanin content and gene expression of *AaLDOX* in fruits injected with EV or 35::*AaLDOX*. The values are the means ± SDs of three replicates. Statistical significance: *P < 0.05; **P < 0.01. **f–h** Phenotype, anthocyanin content and gene expression of *AaLDOX* in fruits injected with pTRV1/pTRV2 and pTRV1/pTRV2::*AaLDOX*. The values are means ± SDs of three replicates. Statistical significance: ***P < 0.001

The *AaLDOX*-encoded leucoanthocyanidin dioxygenase catalyzes the conversion of leucocyanidin to anthocyanidin (Fig. 6a). In our previous study, we discovered that *AaLDOX* is the key structural gene involved in the red coloration of *A. arguta* (Li et al., 2018), but its precise function has not been confirmed. Here, we measured the expression level of *AaLDOX* in *A. arguta* germplasms with red and green colors and detected high expression in red germplasms but low expression in green germplasms, suggesting that *AaLDOX* was positively correlated with fruit color (Fig. 6b). Furthermore, we performed transient overexpression and silencing of *AaLDOX* in *A. arguta*. As expected, overexpression of *AaLDOX* accelerated red pigment accumulation in fruits injected with 35::*AaLDOX* compared with that in fruits injected with only the empty vector as the control (Fig. 6c). The anthocyanin content and expression of *AaLDOX* in the OE-*AaLDOX* fruits were greater than those in the control fruits (Fig. 6d-e). In contrast, red pigment accumulation was inhibited in the fruits injected with pTRV1/pTRV2-*AaLDOX* but normally accumulated in the fruits injected with pTRV1/pTRV2 as the control (Fig. 6f). The anthocyanin content and expression of *AaLDOX* in the silenced fruits were lower than those in the control fruits (Fig. 6g-h). Therefore, we deduced that *AaLDOX* is the key structural gene involved in anthocyanin biosynthesis and that high expression of *AaLDOX* is indispensable for red coloration in *A. arguta*.

### A 29-bp indel variation determines the activity of the *AaLDOX* promoter

**Fig. 7:**
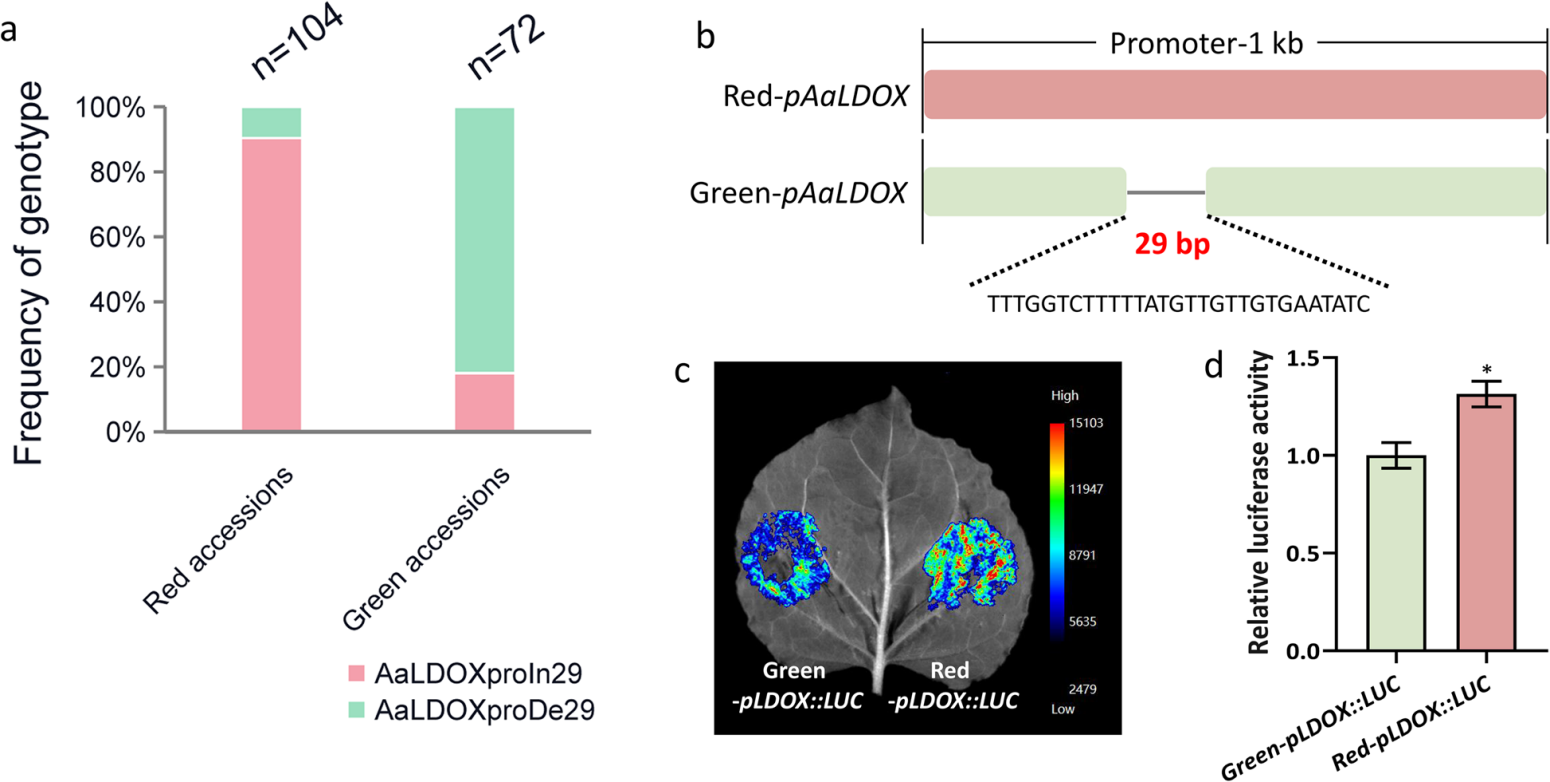
29-bp indel variation analysis. **a** 29-bp indel variation detected in 176 *A. arguta* accessions. **b** Promoter diagram of *AaLDOX* with a 29-bp indel in red and green *A. arguta* cultivars. **c** The activity of the *AaLDOX* promoter with a 29-bp insertion and deletion was analyzed via a luminescence intensity imaging system. Blue and red represent low and high luminescence intensities, respectively. **d** Relative luciferase activity of the *AaLDOX* promoter with a 29-bp insertion and deletion. The values are the means ± SDs of three replicates. Statistical significance: *P < 0.05

In general, differential expression is derived from transcriptional regulation. The significant differences in *AaLDOX* expression between red and green *A. arguta* germplasms (Fig. 6b) promoted us to consider upstream promoter presentation. Through the cloning and analysis of the promoter sequences of 176 *A. arguta* accessions, we identified 43 SNPs and 4 Indels in the *AaLDOX* promoter in red/green *A. arguta* (Fig. S11). Further analysis of the associations between these variations and color phenotypes revealed that the 29-bp indel located 526 bp upstream of the ATG is highly correlated with the red/green trait, which is associated with a 29-bp insertion in most red accessions (94/104), while 29-bp deletion in most green accessions (59/72) (Fig. 7a). A marker developed on this indel was verified in *A. arguta* accessions that met the phenotype requirements (Fig. S12). To investigate the effect of the 29-bp indel on the activity of the *AaLDOX* promoter, the promoter without a 29-bp deletion from the red *A. arguta* cultivar ‘ZHB’ and the green promoter with a 29-bp deletion from the green *A. arguta* cultivar ‘ZLB’ were cloned and inserted into the pGreenII0800 plasmid to perform LUC assays (Fig. 7b). As expected, the luminescence intensity (Fig. 7c) and luciferase activity (Fig. 7d) of AaLDOXproIn29 were greater than those of AaLDOXproDe29, suggesting that the 29-bp indel determines promoter activity and affects *AaLDOX* expression.

## Discussion

Based on genome information and diploid characteristics, the mechanism of red fruit formation has been extensively studied, and several useful markers have been developed to assist in the breeding of fruit plants, including apple trees (Takos et al. 2006; Allan et al. 2008; Li et al. 2012; Zhang et al. 2019; Wang et al. 2022a), pear trees (Yao et al. 2017; Liu et al., 2023), and grape vines (Kobayashi et al., 2004; Jiu et al., 2021). Red color is an important goal in kiwifruit breeding. Red *A. arguta* is an ideal resource for breeding because it is red in both its skin and flesh. Therefore, we first assembled a high-quality reference genome using as the sequencing material ‘Tianyuanhong’, which is completely red when it has ripened. This chromosome-level genome provides the basis for the subsequent RNA-seq in this study (Fig. 1a-c), as well as for other analyses in the future. Comparative genome analysis revealed that the expansion and contraction of gene families play important roles in the formation of *A. arguta* species (Fig. 1d). Based on this genome, we conducted transcriptome analysis in red/green *A. arguta*, and screened a key candidate TF, AaBEE1, involved in anthocyanin regulation in both skin and flesh through differential expression comparison (Fig. 2). Transient overexpression in red tobacco leaves and *A. arguta* fruits and stable genetic transformation in *A. thaliana*, *S. lycopersicum* and *N. tabacum* demonstrated that AaBEE1 inhibited anthocyanin biosynthesis (Fig. 2c, Fig. 3). The color of seed coat in *Arabidopsis* generally caused by proanthocyanidins, but the seed coat color changed after over-expression of *AaBEE1* in *Arabidopsis*. We hypothesized, except for anthocyanin pathway, proanthocyanidin (PA) pathway can also be affected by *AaBEE1*. Anthocyanin and PA biosynthesis are close branches belonged to flavonoid pathway, and these two pathways generally dynamically balance in plants. Previous study in kiwifruit showed transcription factor MYBC1 and WRKY44 are able to regulate balances of anthocyanin and PA (Peng et al. 2019). Similarly, CabHLH regulates PA and anthocyanin regulation in chickpea (Pal et al., 2023), and NtMYB12 is involved in the competition between flavonol and (pro)anthocyanin biosynthesis in *Narcissus tazetta* tepals (Yang et al. 2023). Therefore, we deduced AaBEE1 as the transcription factor not only has a role in regulating anthocyanin but also PA which need to further explore.

**Fig. 8:**
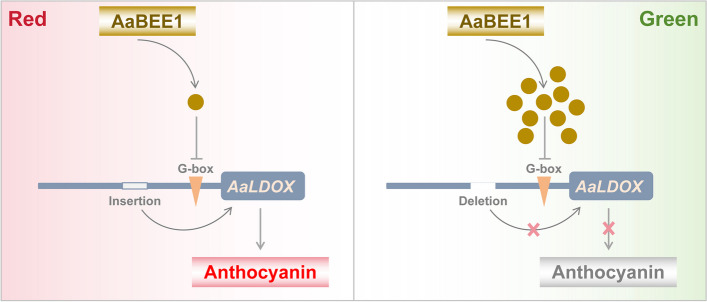
Proposed regulatory model of anthocyanin biosynthesis in *A. arguta*. In red *A. arguta*, less AaBEE1 is produced to bind the G-box of the *AaLDOX* promoter, and the high activity of the *AaLDOX* with a 29-bp insertion leads to high expression of *AaLDOX*, thus inducing the normal biosynthesis of anthocyanin. In green *A. arguta*, more AaBEE1 is produced to bind the G-box of the *AaLDOX* promoter, and low activity of *AaLDOX* with a 29-bp deletion leads to low expression of *AaLDOX*, thus resulting in a barrier to anthocyanin biosynthesis

Furthermore, we demonstrated that AaBEE1 inhibited anthocyanin biosynthesis by directly targeting *AaLDOX* and suppressing its expression via DAP-seq coupled with Y1H, EMSA, Chip-qPCR and LUC assays (Fig. 4, Fig. 5), which is consistent with other reports that TFs play a role generally by targeting structural genes (Liu et al. 2017; Wang et al. 2022a). *AaLDOX* encodes leucoanthocyanidin dioxygenase, which catalyzes the conversion of leucocyanidin to anthocyanidin (Fig. 6a). *AaLDOX* expression is indispensable for anthocyanin biosynthesis (Fig. 6b-h). Deliberate control of gene expression is usually derived from promoter regulation (Pope and Medzhitov 2018). Promoter cloning and analysis revealed a 29-bp indel that was highly correlated with the red/green phenotype (Fig. 7a), and this indel induced differential activity of the *AaLDOX* promoter (Fig. 7b-d). Therefore, we proposed a regulatory model in which, in red *A. arguta* without deletion of the 29-bp indel, less AaBEE1 binds the *AaLDOX* promoter to suppress its activity, resulting in the high activity, normal expression, and participation in anthocyanin biosynthesis. In contrast, in green *A. arguta* with deletion of the 29-bp indel, more AaBEE1 binds the *AaLDOX* promoter to suppress its activity, resulting in low activity, abnormal expression, and no participation in anthocyanin biosynthesis (Fig. 8). Although AtBEE1 negatively regulates anthocyanin synthesis in *Arabidopsis* (Petridis et al. 2016), few studies have revealed the function of BEE1 in anthocyanin regulation in fruit trees. Therefore, the discovery that AaBEE1 regulates fruit color both in the skin and flesh of *A. arguta* provides an important reference for BEE1 functional identification in other fruit trees. Previous studies have focused on the influence of positive regulatory factors on anthocyanin biosynthesis in kiwifruit. The discovery of AaBEE1 will provide novel opportunities for exploring more negative regulatory factors and understanding their molecular mechanism. It is easy to think that MYB or MBW complexes are generally served as the core control hub positively regulating anthocyanin biosynthesis when it comes to the issue of red fruit color, such as the key role of MYB110 (Peng et al. 2019) and MYB123/bHLH42 (Wang et al. 2019) in kiwifruit anthocyanin regulation. In this study, the identification of BEE1 as a bHLH transcription factor that negatively regulates anthocyanin biosynthesis not only broaden the anthocyanin regulation study but also provide a direct target in gene editing-mediated color breeding in the future. Additionally, a genome comparison of ‘Tianyuanhong’ and ‘LC2’ revealed larger TEs in red ‘Tianyuanhong’ than in green ‘LC2’, suggesting that TEs might play roles in anthocyanin regulation in *A. arguta,* as has been reported in other fruit species, including red apple (Zhang et al. 2019) and orange (Huang et al. 2018). Further studies will focus on this point.

*LDOX*, a key structural gene in the anthocyanin biosynthetic pathway, has been reported to determine red color formation in other fruit trees. Insertion in the coding region of *PgLDOX* results in ‘white’ anthocyanin-less pomegranate (*Punica granatum* L.) (Ben-Simhon et al. 2015). In *A. arguta*, color-related variations were not detected in the coding region of *AaLDOX* (data not shown), indicating that fruit color is not associated with CDS variation. More often, the differential expression of *LDOX* is regulated by TFs (Karppinen et al. 2021; Zhang et al. 2022b). Differential expression of *AcLDOX*, which is targeted by AcbHLH42, is involved in anthocyanin regulation in the flesh of *A. chinensis var. chinensis* ‘Hongyang’ (Wang et al. 2019). In this study, *AaLDOX* was differentially expressed between red and green samples (Fig. 6b), which prompted us to clone the promoter of *AaLDOX.* Based on this assembled genome, we easily obtained precise sequences in 176 accessions and found a 29-bp indel variation tightly associated with the red/green phenotype and developed it as an indel marker for color phenotype (Fig. 7a, Fig. S12). Using this indel marker, we identified male *A. arguta* accessions in our germplasm repository to assist in the selection of male parents for kiwifruit breeding (Fig. S13). The color of the skin and flesh of *A. arguta* can be divided into three types: fruit type 1, with red skin and red flesh (most red *A. arguta* fruits are this type); fruit type 2, with red skin and green flesh (few *A. arguta* fruits are this type); and fruit type 3, with green skin and green flesh (most green *A. arguta* fruits are this type) (Fig. S14). There were some accessions whose fruit color did not correspond to the expected indel variation, such as ‘R77: 6–22–47’, ‘R78: 6–22–76’ and ‘R79: 6–23–73’, with light red skin but heterozygous indel alleles. Through further analysis, we found that these three *A. arguta* accessions belong to fruit type 2, with red skin and green flesh (Fig. S15), indicating that their differences in skin and flesh coloration can be explained by the comprehensive expression profiles of all characterized anthocyanin-related genes, including biosynthetic genes and regulators whose expression, from the RNA-seq data, could be divided into two clusters, skin and flesh (Fig. S16). This result is consistent with previous studies in other fruit trees in which the coloring mechanisms differ between fruit tissues (Espley et al. 2009; Zhang et al. 2019; Castillejo et al. 2020). Additionally, we found dose differences of this indel between accessions by analyzing the band intensity via SDS-PAGE. Phenotype is affected by gene dosage after genome doubling (Zhang et al. 2010; Guo et al. 2013). As a tetraploid plant, *A. arguta* has experienced a genome doubling event (Huang et al. 2013), so gene dosage effects might be prevalent in *A. arguta*. The G-box in *AaLDOX* promoter targeted by AaBEE1 is not directly located within the indel variation. Similarly, in tomato, a 27-bp indel in the promoter of *SlBBX31* is associated with cold tolerance, but the related ACE motif is not located within the 27-bp indel and is bound by upstream regulators to control *SlBBX31* expression (Zhu et al., 2023). Based on the inhibition of AaBEE1 to *AaLDOX* promoter, AaBEE1 appears to be more crucial in regulating anthocyanin biosynthesis. The potential upstream regulatory factor of AaBEE1 might involve in this regulation which need to be further explored.

## Conclusion

A high-quality chromosome-level genome of ‘Tianyuanhong’ was assembled, and comparative genome analysis revealed a role of expansion/contraction of gene families in the formation of species-specific traits in *A. arguta*. Through a series of molecular experiments, we confirmed that AaBEE1 negatively regulates anthocyanin biosynthesis by directly targeting the *AaLDOX* promoter, in which a 29-bp indel associated with fruit color was identified, and an indel marker was also developed for use in identifying male *A. arguta* with potential color phenotypes.

## Materials and methods

### Genome sequencing, assembly and annotation

*A. arguta* cv. ‘Tianyuanhong’ grown at the Zhengzhou Fruit Research Institute, Chinese Academy of Agricultural Sciences (Zhengzhou, Henan Province) was used for genome assembly. Fresh young leaves were collected for genomic DNA extraction, library construction and genome sequencing, including BGI, PacBio HiFi and Hi-C sequencing. The genome size of *A. arguta* was estimated via k-mer analysis by Jellyfish v2.2.10 (Marçais and Kingsford 2011) and GCE v1.0.2 (Liu et al. 2013). PacBio HiFi reads were de novo assembled using Hifiasm v0.19.5 with parameter -l3 –primary –n-hap 4 (Cheng et al. 2021). A Hi-C interaction map was used to check and correct potential misassemblies. The quality of the genome assembly was evaluated using BUSCO (Manni et al. 2021) and LAI assessments (Ou et al. 2018). Different types of annotations, including repeat annotation, gene annotation and functional annotation, were conducted for the *A. argut*a genome. Repeat libraries were constructed via RepeatModeler v2.0.1 (Flynn et al. 2020) and LTR-FINDER v1.0.7 with setting parameter -w 2 -C (Xu and Wang 2007) and then utilized to scan the assembled genome with RepeatMasker v4.1.2 with setting parameter -nolow -no_is -norna -parallel 2 (Chen et al., 2004). The protein-coding genes were predicted via homology-based prediction using Exonerate v2.4.0 (Slater and Birney 2005), transcriptome-based prediction using PASA v.2.4.1 (Haas et al. 2003) and de novo prediction using Augustus 3.4.0 (Stanke., 2004, 2005, 2006) and GlimmerHMM v3.0.4 (Majoros et al. 2004). All these predictions were integrated via MAKER v3.01.03 with default parameters to obtain a consensus set of gene models (Cantarel et al. 2008). Gene functions were inferred according to the best match of alignments to NCBI, NR, KEGG, GO, TrEMBL, and Swiss-Prot via Diamond BLASTP v2.0.7 (Buchfink et al. 2021). Genome relevant sequence data has been submitted in the public database NGDC (National Genomics Data Center) with the Bioproject ID PRJCA033191.

### Annotation of non-coding RNAs

In accordance with the structural features of tRNAs, tRNAscan-SE software was used to search for tRNA sequences in the genome (Lowe and Eddy 1997). RNAmmer software version 1.2 was used to predict rRNA sequences in the genome (Lagesen et al. 2007). The miRNAs and snRNAs were predicted using INFERNAL software in the Rfam database (Griffiths-Jones et al. 2005; Nawrocki and Eddy 2013).

### Construction of the phylogenetic tree

The genes of single-copy orthologous gene families were aligned using MUSCLE software (Edgar 2004). A phylogenetic tree was constructed using RAxMl software via the maximum likelihood method (Stamatakis 2014).

### Comparative genomic analysis

A total of 12 plant species were selected to identify clusters of gene families, including nine *Actinidia* species (*A. latifolia*, *A. eriantha*, *A. chinensis*, *A. hemsleyana*, *A. rufa*, *A. polygama*, *A. arguta* ‘LC2’, *A. arguta* ‘M1’, *A. arguta* ‘Tianyuanhong’) and three other species (*Vitis vinifera*, *Solanum lycopersicum* and *Camellia sinensis*). Combined with the time correction points obtained via the TimeTree website (Hedges et al., 2006) and the relevant literature, the divergence times were estimated via r8s (Sanderson 2003) and the MCMCtree program in PAML (Yang 1997). Based on the cluster analysis results of gene families, the expansion and contraction of gene families were analyzed via CAFE software (De Bie et al., 2006). The significant expansion and contraction of gene family members were determined via GO/KEGG enrichment.

### Plant materials for experiments

‘ZHB’ and ‘ZLB’ were grown at Xinxiang Experimental Station of Zhengzhou Fruit Research Institute in Henan Province. *A. thaliana* ‘Col-0’, *Solanum lycopersicum* ‘Micro-Tom’ and *Nicotiana tabacum* ‘SR1’ were used for stable genetic transformation. Red-background tobacco (stably transformed peach MYB TF PpMYB75: Prupe.3G163100) leaves and *A. arguta* fruits were used for transient overexpression of *AaBEE1*. Two tetraploid *A. arguta* cultivars, ‘ZHB’ with a red color in both the skin and flesh when the fruit ripens and ‘ZLB’ with a green color in both the skin and flesh when the fruit ripens, were selected for RNA-seq based on the ‘Tianyuanhong’ genome. At three different stages (S1, green stage; S2, color-changing stage; and S3, red stage), fruits of the red *A. arguta* cultivar ‘ZHB’ and the green *A. arguta* cultivar ‘ZLB’ were collected and used for RNA-seq. RNA sequencing data has been uploaded to public database NGDC (National Genomics Data Center) with the accession number CRA020742.

### Phylogenetic analysis

The protein sequences of AaBEE1 and of bHLH TFs reported in different plant species to be involved in anthocyanin regulation were used to construct a phylogenetic tree using MEGA 6.0 (Tamura et al. 2013) via the neighbor-joining method with 1000 bootstrap replications. The tree was visualized using the online tool EVOLVIEW (Zhang et al. 2012). Protein domains were identified via SMART (Letunic et al., 2020).

### Vector construction

The full-length coding sequence (CDS) of *AaBEE1* was amplified from red *A. arguta* ‘Tianyuanhong’ cDNA via specific primers carrying homologous arms and subsequently cloned and inserted into a pBI121-overexpressing vector harboring a GFP tag and kanamycin screening resistance. The recombinant plasmid AaBEE1-pBI121 was then transformed into *Agrobacterium tumefaciens* strain GV3101. The primers used were are showed in Table S10.

### Subcellular localization

The full-length CDS of *AaBEE1* without a stop codon was amplified from red *A. arguta* ‘Tianyuanhong’ cDNA via specific primers with the restriction enzyme sites *Pst* I and *Bam* HI and subsequently cloned and inserted into the 16,318-hGFP vector carrying a GFP tag. Five to seven true leaves of *A. thaliana* under good growth conditions before bolting were used to isolate protoplasts as previously described (Zhai et al. 2009). Subcellular localization was carried out via polyethylene glycol (PEG)-mediated transformation. Fluorescence was observed at excitation wavelengths of 405 nm and 488 nm. The primers used are showed in Table S10.

### Stable genetic transformation in *A. thaliana*, *S. lycopersicum *and *N. tabacum*

The recombinant plasmid constructed above was subsequently transformed into wild-type *A. thaliana* ‘Col-0’ via the floral dip method as described previously (Clough and Bent 1998; Bent 2006). The successfully overexpressed lines were detected by PCR with primers for the kanamycin-resistance gene *NPT*. T2-generation seeds of the OE and WT lines were first subjected to low-temperature stratification and disinfection procedures and then cultured on MS media for one week in a light incubator.

*S. lycopersicum* ‘Micro-Tom’ transgenic lines were obtained via *Agrobacterium*-mediated cotyledon explant transformation as described previously (Park et al. 2003). The successfully overexpressed lines were detected by PCR with primers specific for the resistant gene *NPT*II.

The *N. tabacum* cultivar ‘SR1’ was selected as a transgenic material for stable genetic transformation for the verification of *AaBEE1* function via an *Agrobacterium*-mediated leaf disc transformation as described previously (Cong et al. 2014). The flower color is red in WT ‘SR1’ tobacco, so flower color changes indicated transgenic plants.

### Transient overexpression in tobacco leaves and *A. arguta* fruits

Transient overexpression of *AaBEE1* in tobacco leaves was performed via *Agrobacterium*-mediated infiltration according to existing methods (Li et al. 2020). Transient overexpression of *AaBEE1* and *AaLDOX* in *A. arguta* was carried out via *Agrobacterium*-mediated fruit infiltration according to previous methods (Li et al. 2020). The fruit skin of the ‘Sadowa’ variety 110 days after full bloom, when the fruit is slightly red, was used for the injection of OE-*AaBEE1*. The fruit skin of the ‘Sadowa’ variety 80 days after full bloom, when the fruit is green, was used for the injection of OE-*AaLDOX*. Red-background tobacco materials were a gift from Prof. Zhenhua Lu’s laboratory. Fruits injected with the empty vector were used as the control. Approximately 30 fruits were used for infection per test. Three biological replicates were conducted for each assay. The primers used are shown in Table S10.

### Virus-induced gene silencing (VIGS) in *A. arguta*

VIGS experiments of *AaLDOX* in *A. arguta* were carried out via *Agrobacterium*-mediated fruit infiltration according to previous description (Li et al. 2020). The fruit skin of the ‘Sadowa’ variety 80 days after full bloom, when the fruit is green, was used for injection. Fruits injected with the empty vector (pTRV1 + pTRV2) were used as the control. Approximately 30 fruits were used for infection per test. Three biological replicates were conducted for each assay. The primers used are shown in Table S10.

### DAP-seq (DNA affinity purification sequencing) analysis

DAP-seq analysis was conducted according to previous studies (O'Malley et al. 2016; Bartlett et al. 2017). Briefly, *A. arguta* genomic DNA was isolated and added to an affinity-purified AaBEE1 protein linked to an affinity tag, introduced into the expression vector and purified via MICH DNA Clean Beads, and the unbound gDNA was washed away. The binding portion was eluted and amplified via PCR. MACS was used to call peaks, which were annotated using ChIPseeker (Zhang et al. 2008; Yu et al. 2015). The core motif was identified via MEME-ChIP (Machanick and Bailey 2011). Genes corresponding to the detected peaks were identified and used for GO and KEGG classification analysis.

### Y1H assay

The ~ 1-kb *AaLDOX* promoter was cloned from ‘ZLB’. The ORF of *AaBEE1* and the promoter of *AaLDOX* were cloned and inserted into the pGADT7 and pHIS2 vectors, respectively. In accordance with previous methods (Liu et al. 2022b), pGADT7-*AaBEE1* and pHIS2-*AaLDOXpro* plasmids were cotransformed into the yeast strain Y187; and pGADT-53 and pHIS2-p53 were used as positive control, pGADT7 and pHIS2-p53 were used as negative controls, and pGADT7 and pHIS2-*AaLDOXpro* were used for self-activating detection. The primers used for Y1H are listed in Table S10.

### LUC assay

For the LUC assay, the ~ 1-kb *AaLDOX* promoter was amplified from ‘ZLB’, cloned and inserted into the linearized double-reporter *pGreenII 0800-LUC* vector to form the reporter *AaLDOXpro::LUC* (Hellens et al. 2005), which was subsequently transformed into *A. tumefaciens* strain GV3101 with pSoup. The effector *35Spro::AaBEE1* was transferred into *A. tumefaciens* strain GV3101. *A. tumefaciens* carrying the effector/reporter was suspended in infiltration buffer supplemented with 10 mM MES, 10 mM MgCl2 and 150 mM AS. The effector and reporter were mixed at a 5:1 volume ratio and coinjected into 4–5-week-old *N. benthamiana* leaves as described previously (Gao et al. 2020; Hu et al. 2021; Liu et al. 2022a, b). *35Spro::AaBEE1* and *pGreenII 0800-LUC* alone were also infiltrated into *N. benthamiana* leaves as control. After 2–3 days of infiltration, promoter activities were investigated via fluorescence imaging (LASER900, BIO-OI, Guangzhou, China). At least three biological replicates were performed for each combination. Promoter activity assays of the red/green *AaLDOX* promoter without/with deletion were also conducted. The primers used are shown in Table S10.

For the dual-luciferase reporter assay, the CDS of AaBEE1 was cloned and inserted into the *pGreenII* 62-SK vector, and the 2-kb upstream promoter sequence of *AaLDOX* ATG was cloned and inserted into the *pGreenII 0800-LUC* vector, after which the recombinant plasmids were transformed into *A. tumefaciens* strain GV3101 with pSoup. *Agrobacterium* cells harboring AaBEE1 and *AaLDOXpro* were mixed at a 4:1 ratio (v/v) and injected into 4–5-week-old *N. benthamiana* leaves using a needleless single-use syringe. The infiltrated area of the leaves was sampled to detect LUC activity via the Dual-Luciferase® Reporter Assay System (E1910; Promega). The LUC/REN ratio of each experimental sample was normalized relative to that of the control sample. The experiments were carried out in triplicate. The primers used are shown in Table S10.

### Electrophoretic mobility shift assays (EMSA)

For EMSA, the AaBEE1 plasmid protein was first expressed in prokaryotic cells. Probes labeled with FAM via 5’-fluorescein phosphoramidite kit containing natural and mutant binding sites were specifically synthesized by Sangon Biotech. A cold probe without labels was used as the competitor. The probes and purified AaBEE1 protein were coincubated in binding buffer for 35 min, after which the reaction mixtures were loaded onto a 6% nondenatured polyacrylamide gel and subjected to electrophoresis at 100 V for 1 h at 4 °C. The gel was electroblotted onto a nylon membrane in 0.5 × TBE buffer at 300 mA for 30 min. Membrane washing and signal detection were performed using the LightShift® Chemiluminescent EMSA Kit (Thermo Fisher Scientific, MA, USA) according to the manufacturers’ instructions and the Gel Doc2000 imaging system (Bio-Rad, USA). The primers used are shown in Table S10.

### Chromatin immunoprecipitation followed by quantitative PCR (Chip-qPCR)

*A. arguta* leaves overexpressing AaBEE1-GFP fusions were used for ChIP according to previous methods (Zong et al. 2016; Zheng et al. 2022). Briefly, approximately 3 g of *A. arguta* leaves infiltrated with 35S::AaBEE1-GFP were harvested at ZT 12 h and cross-linked in 1% (v/v) formaldehyde for 10 min under vacuum conditions. The chromatin mixture was sonicated to obtain 200–1000-bp DNA. Immunoprecipitation reactions were carried out with an anti-GFP antibody (ab290). DNA was purified via a PCR purification kit (QIAGEN). Chip-qPCR was performed to detect the relative enrichment via the percent input method. The primers used are shown in Table S10.

### Promoter analysis

The promoter of *AaLDOX* was cloned from the red and green genomic DNA of *A. arguta* cultivars via homologous cloning with specific primers. A sequence with a length of approximately 1 kb could be successfully amplified and inserted into the T-vector via the TA cloning method. The fusion vector was transformed into *Escherichia coli* DH5α competent cells that were cultured on LB agar plates with ampicillin and incubated at 37 °C for 16 h. At least 20 positive clones were sequenced via the Sanger system (SunYa Biotech, Shanghai, China). Promoter variations were detected and analyzed via MEGA 6.0 (Tamura et al. 2013).

### Identification of red/green germplasms via indel marker

Based on the 29-bp deletion in the promoter of *AaLDOX*, specific indel primers were designed with Primer 5. A total of 25 µL of the reaction system (12.5 µL 2 × T5 Super mix PAGE, 1 µL forward primer, 1 µL reverse primer, and 1 µg gDNA, ddH_2_O up to 25 µL) was amplified with the PCR procedure set at 94 °C for 3 min; 35 cycles of 94 °C for 30 s, 58 °C for 30 s, and 72 °C for 30 s; and 72 °C for 10 min of terminal extension. Amplified DNA fragments were separated via denaturing PAGE (polyacrylamide gel electrophoresis) and visualized via silver staining after fixation, penetration and rinsing.

### Anthocyanin measurement

The anthocyanin content of different plant samples was determined via a Plant Anthocyanin Content Assay Kit (boxbio, Beijing, China) following the manufacturer’s instructions. The detection wavelengths of the absorbance value of the extraction solution were set at 530 nm and 700 nm. The anthocyanin content was calculated via the specific formula provided in the kit.

### Quantitative real-time PCR (qRT-PCR)

Total RNA was isolated via a the Quick RNA Isolation Kit (Huayueyang Biotechnology Co., Ltd, Beijing, China) according to the manufacturer’s instructions.The quality and integrity of the RNA samples were assessed via 1% agarose gel electrophoresis and a NanoDrop 2000 micro-ultraviolet spectrophotometry (Thermo Fisher Scientific, MA, USA), respectively. First-strand cDNA was synthesized via ReverTra Ace qPCR RT Master Mix FSQ-201 (TOYOBO, Osaka, Japan) according to the manufacturer’s protocol. A total of 20 µL of reaction mixture for qRT-PCR containing 10 µL of qPCR Mix, 1 µL of forward or reverse primer, 3 µL of cDNA template, and 5 µL of ddH2O was run on a LightCycler® 480 real-time PCR system with a 96-well plate under the following PCR conditions: 95 °C for 5 min, followed by 45 cycles of 95 °C for 10 s, 60 °C for 20 s, and 72 °C for 20 s, after which a melting curve was obtained by using the default parameters of 95 °C for 5 s and 65 °C for 1 min. PCRs were performed for three biological replicates. Relative expression levels were calculated via the 2^−ΔΔCt^ method (Vandesompele et al., 2002). The primers used are shown in Table S10.

### Statistical analysis

Statistically significant differences between two conditions were determined via Student’s t test at *P* < 0.05. All analyses were performed via GraphPad Prism 8 software (GraphPad Software Inc., San Diego, CA, USA). For qRT-PCR and anthocyanin content, the data are presented as the means ± SE of three biological replicates.

## Supplementary Information

Additional file 1: Fig. S1. The assembled 29 chromosomes. One color (black or gray) represents a continuous sequence (contig). Among 29 chromosomes, 20 have no gaps. Fig. S2. K-mer frequency analysis to estimate the *A. arguta* genome. a The *A. arguta* k-mer result. b-d The k-mer result of reported autotetraploid *R. officinale*, *S. tuberosum* and *M. sativa*. The peak at location a refers to high heterozygosity, and the peak at 4a refers to autotetraploidy. Fig. S3 Gene ontology (GO) enrichment and Kyoto Encyclopedia of Genes and Genomes (KEGG) pathway enrichment for expanded and contracted gene families of ‘Tianyuanhong’. a GO enrichment of expansion family. b GO enrichment of contraction family. c KEGG enrichment of expansion family. d KEGG enrichment of contraction family. The vertical axis represents the specific biological pathways, and the horizontal axis represents the number of genes involved in the biological pathways. Fig. S4 Venn diagram of DEGs among different comparisons. a All DEGs with 2-foldchange among six comparisons. b All up-DEGs with 2-foldchange among six comparisons. c Down-DEGs among three comparisons, including 160 common down-DEGs, down-DEGs with 8-foldchange in ZLB-Skin-S3 vs. ZHB-Skin-S3, and down-DEGs with 8-foldchange in ZLB-Flesh-S3 vs. ZHB-Flesh-S3. Fig. S5 Phylogenetic analysis and amino acid sequence alignment of AaBEE1 and other anthocyanin biosynthesis-related IIIf subgroup bHLH transcription factors from other plant species. Phylogenetic tree was constructed using neighbor-joining method. Specific bHLH protein accession numbers were generated from NCBI or Genome database and showed below: FabHLH3 (AFL02463), MdbHLH3 (ADL36597), LjTT8 (AB490778), MtTT8 (KM892777), VvMYC1 (ACC68685), AcbHLH42 (QAT77714), NtAN1b (HQ589209), NtAN1a (HQ589208), PhAN1 (AAG25928), StAN1 (JX848660), SlAN1 (Solyc09g065100), LhbHLH2 (BAE20058), OsRc (ABB17166), AtTT8 (Q9FT81), LjGL3 (AB492284), NtJAF13a (KF305768), NtJAF13b (KF298397), StGL3-like (NM_001288203), SlJAF13 (Solyc08g081140), PhJAF13 (AAC39455), ZmR (P13027), AtEGL1 (Q9CAD0), AtGL3 (NP_680372), VvMYCA1 (ABM92332), FvEGL1 (XP_004308377), MdbHLH33 (ABB84474), AtbHLH061 (AAM10950), AtbHLH116 (AAL84972), AtDYT1 (O81900), AtbHLH021 (NP_179283), AtbHLH027 (AAS79544), AtbHLH035 (NP_974948), AtJAM2 (Q9LNJ5), AtbHLH017 (AAM19778), AtMYC2 (Q39204), AtbHLH028 (AAL55721), AtBEE1 (NP_173276). Fig. S6 Relative expression level of *AaBEE1* in ‘ZHB’ and ‘ZLB’. Fig. S7 Relative expression level of anthocyanin biosynthetic genes (*AaPAL, AaC4H, Aa4CL*) in OE-*AaBEE1* and EV. a Relative expression of AaPAL. b Relative expression of AaC4H. c Relative expression of *Aa4CL*. Values are means ± SD for three replicates. Statistical significance: *P < 0.01; **P < 0.01; ***P < 0.001. Fig. S8 Overexpression of AaBEE1 in *A. thaliana*. a Anthocyanin content in WT and transgenic lines. b Expression of AaBEE1 in WT and transgenic lines. Values are means ± SD for three replicates. Statistical significance: ***P < 0.001. Fig. S9 Relative expression level of anthocyanin biosynthetic genes in transgenic materials. a Relative expression level of anthocyanin biosynthetic genes in transgenic *Arabidopsis*. b Relative expression level of anthocyanin biosynthetic genes in transgenic tomato. Values are means ± SD for three replicates. Statistical significance: ***P < 0.001. Fig. S10 Relative expression level of *AaBEE1* and anthocyanin biosynthetic genes in transgenic tobacco. a Relative expression of *AaBEE1*. b Relative expression of *AaPAL*. c Relative expression of *Aa4CL*. d Relative expression of *AaCHS*. e Relative expression of *AaF3H*. f Relative expression of *AaLDOX*. Values are means ± SD for three replicates. Statistical significance: **P < 0.01; ***P < 0.001. Fig. S11 Variation analysis of *AaLDOX* promoter. 43 SNPs and 4 Indels variation were obtained. Fig. S12 Indel marker verification in 176 natural populations of *A. arguta* with red/green color by SDS-PAGE. One/two bands indicate that the marker is homozygous/heterozygous in the samples. Fig. S13 Red/green identification of male *A. arguta* based on the indel marker. Fig. S14 Three typed *A. arguta* with different color in skin and flesh. Fruit type 1 with red skin and red flesh; fruit type 2 with red skin green flesh; fruit type 3 with green skin green flesh. Fig. S15 Three fruit type 2 *A. arguta*. Fig. S16 The comprehensive expression profiles of anthocyanin biosynthetic genes including biosynthetic genes and its regulators in RNA-seq.

Additional file 2: Table. S1. Statistics of the assembled *A. arguta* genome and previously published *Actinidia* genomes. Table S2. Statistics of annotation of gene function. Table S3. Statistics of repeat sequences in 'Tianyuanhong' genome. Table S4. Statistics of different typed transposons in 'Tianyuanhong' genome. Table S5. Statistics of annotation of non-coding RNAs. Table S6. Specific regions of identified centromere and telomere. Table S7. Statistics of gene families in different species. Table S8. DEG analysis in transcriptome data. Table S9. The screening of candidate genes. Table S10. Primers used in this study.

## Data Availability

The authors confirm that all data in the study are included this article (and its supplementary information file).

## Abbreviations

CDS: Coding sequence
Chip-qPCR: Chromatin immunoprecipitation followed by quantitative PCR
DAP-seq: DNA affinity purification sequencing
DEG: Differentially expressed genes
EMSA: Electrophoretic mobility shift assays
GFP: Green fluorescent protein
GO: Gene ontology
KEGG: Kyoto encyclopedia of genes and genomes
LAI: LTR assembly index
LTR: Long terminal repeat
LUC: Luciferase
NGDC: National genomics data center
OE: Overexpression
PA: Proanthocyanidin
PAGE: Polyacrylamide gel electrophoresis
PEG: Polyethylene glycol
PSA: Pseudomonas syringaepv. Actinidiae
qRT-PCR: Quantitative real-time PCR
TF: Transcription factor
TSS: Transcription initiation site
UTR: Untranslated region
VIGS: Virus-induced gene silencing
Y1H: Yeast one-hybrid
